# The impact of high school athletic trainer services on medical payments and utilizations: a microsimulation analysis on medical claims

**DOI:** 10.1186/s40621-019-0194-y

**Published:** 2019-05-01

**Authors:** Tao Li, Samuel T. Johnson, Michael C. Koester, Annie Hommel, Marc F. Norcross

**Affiliations:** 1 Health Management and Policy Program, School of Social and Behavioral Health Sciences, College of Public Health and Human Sciences, Oregon State University, Corvallis, Oregon USA; 2 Kinesiology Program, School of Biological and Population Health Sciences, College of Public Health and Human Sciences, Oregon State University, Corvallis, Oregon USA; 3 Slocum Center for Orthopedics and Sports Medicine, Eugene, Oregon USA

**Keywords:** Athletic trainer, Medical claims, Microsimulation, Medicaid, Commercial insurance, Medical payments, Emergency department visits

## Abstract

**Background:**

Increasing athletic trainer (AT) services in high schools has attracted widespread interest across the nation as an effective instrument to manage injuries and improve children’s health, but there is a lack of evidence on potential medical savings. Our study aimed to address this knowledge gap and provide evidence of AT impacts on medical payments and utilizations to inform public policy decision.

**Methods:**

We obtained medical claims of patients aged 14 to 18 years from the 2011–2014 Oregon All Payer All Claims limited dataset. We calculated payer payments and utilizations for medical claims under AT’s scope of practice. We used zip codes to link patients with the enrollment boundaries of Oregon public high schools, which were classified as either “AT group” or “non-AT group”. We implemented an innovative microsimulation analysis to address the uncertainty of linkage between children and schools.

**Results:**

Our analysis included 64,115 and 84,968 eligible children with Medicaid and commercial insurance, respectively. Associated with high school AT services, Medicaid saved an average of $64 per patient during the study period, while commercial insurance payment rarely changed. AT services may reduce emergency visits for both insurance types but increase total visits for commercially insured patients.

**Conclusions:**

Our study provides evidence for the differential impacts of AT services on medical payments and utilizations. The legislators should consider to allocate funds for high schools to directly employ ATs. This will encourage ATs to work to their highest ability to improve children’s wellbeing while containing avoidable medical cost.

## Background

Athletic trainers (AT) are health care professionals that work in collaboration with physicians to provide a range of services, including injury and illness prevention, evaluation, treatment, and rehabilitation as well as emergency care, to physically active indiviudals (Pierpoint et al. 2018; Johnson et al. 2017; Kroshus et al. 2017; Yard et al. 2009; Kerr et al. 2016; Hambleton et al. 2012). Athletic trainer services can also improve children’s health at the population level. For example, several recent studies provide evidence that schools with an AT show a greater recognition of concussions (Pierpoint et al. 2018; Kroshus et al. 2017), lower musculoskeletal injury and re-injury rates (Pierpoint et al. 2018), and are more likely to have athletics-specific emergency action plans (Johnson et al. 2017). The need for AT services among active adolescents was emphasized by the American Medical Association, calling for AT services in all high schools with sports programs (Lyznicki et al. 1999). More recently, the American Academy of Pediatrics’ Council on Sports Medicine and Fitness (2015), the National Federation of State High School Associations (n.d.), and the Appropriate Medical Care for Secondary School-Aged Athletes Task Force (Almquist et al. 2008) recommend that an AT be available to provide medical care for secondary school-aged athletes. However, while these recommendations are based on “ensuring that young athletes receive consistent and adequate medical care while participating in practices and games” (Almquist et al. 2008), they do not consider the cost of providing AT services. Therefore, it is not surprising that national estimates reveal that only 70% of high schools provide some level of AT services (ranging from a set number of hours per sport season to full-time), and that access to ATs varies greatly across states (Pryor et al. 2015).

By improving children’s health, AT services in high school may offer the potential to reduce medical payments and utilizations. However, there is still a lack of evidence on potential medical savings in the literature. In a preliminary analysis using the Oregon All Payer All Claims (APAC) data, Li et al. was not able to conclusively determine whether there is an association between AT services and medical savings (Li et al. 2018). Li et al. attributed the inconclusive results to data limitations of the APAC data that required the use of zip codes to link patients’ medical claims with the enrollment boundaries of Oregon public high schools. However, as a zip code may overlap the enrollment boundaries of multiple high schools with different AT availability status, this caused uncertainty when linking patients to schools that had no ATs (the “non-AT group”) or to schools that had ATs (the “AT group”). In the previous exploratory study, the data limitation was addressed by only including zip codes in which all schools within the zip code had periods of time when AT services were available and periods of time when AT services were unavailable (Li et al. 2018). While this method allowed Li et al. to aggregate all claims within zip codes into the same AT availability status and then conduct paired *t*-tests at the zip code level to estimate potential savings, such strict inclusion criteria resulted in the inclusion of less than 10% of total zip codes in the state and less than 2% of total patients aged 14–18 with AT-relevant claims. Given that about half of high schools in Oregon had AT services (Pryor et al. 2015), analysis based on a small portion of the study population may have been insufficient to fully capture the impact of AT services at the state level. Therefore, the purpose of this current study was to implement an innovative microsimulation analysis to provide a more comprehensive assessment of the potential impact of AT services in high schools on medical payments and utilizations. We expect this study to inform evidence-based public policy decisions on expanding high school AT services.

## Methods

### Data

To study the impact of AT services on medical payments and utilizations among high school students, our main data source was medical claims of patients aged 14 to 18 years from the 2011–2014 APAC limited dataset. Operated by the Oregon Health Authority, the APAC include administrative healthcare data for Oregonians who are insured through commercial insurance, Medicaid, and Medicare (Oregon All Payer All Claims Database n.d.). However, based on the APAC dataset, we lacked information to examine which high school a patient in this age group attended. To address this limitation, we used zip codes from medical claims in the APAC dataset to link patients with the enrollment boundaries of Oregon public high schools, which were then classified as either having or not having AT services using data from the Oregon Athletic Trainers’ Society.

### Measure of payments and utilizations

Prior to data analysis, two certified athletic trainers (M.F.N., S.T.J.) and one pediatric sports-medicine physician (M.C.K.) achieved consensus on which International Classification of Diseases, 9th edition (ICD9) and Current Procedural Terminology (CPT) codes were under ATs’ scope of practice in Oregon. For each patient, we included any visits when both the primary diagnosis and the primary procedure were related to ATs’ practice scope. Patients with these AT-relevant claims consisted of our study population. Our recent study (Li et al. 2018) suggested that the presence of AT services in high schools may have different impacts on medical payments by Medicaid and commercial insurance. Therefore, we analyzed payer payments separately for Medicaid and commercial insurance in this study. In addition, to adjust for the different number of patients between the AT and non-AT groups, we calculated payer payments per patient rather than the total amount. We also examined the impact of AT services on utilizations in terms of emergency visits and total visits for AT-relevant claims.

### Statistical analysis

**Fig. 1:**
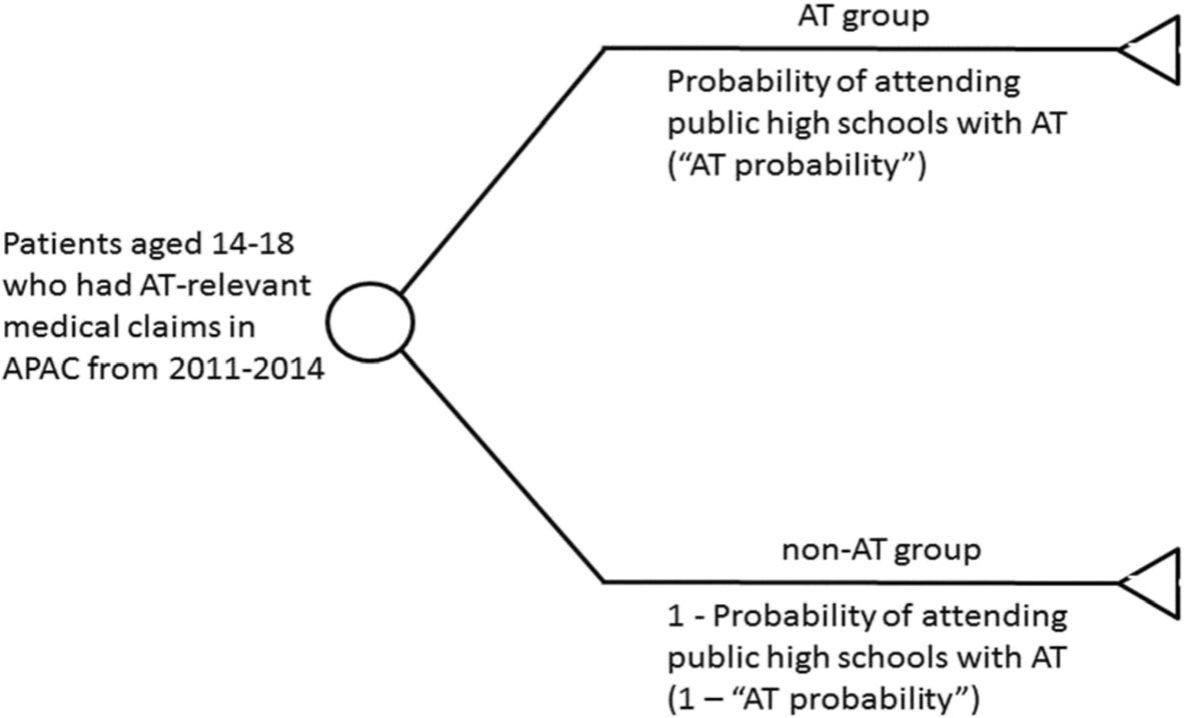
This figure shows the conceptual model of using microsimulation to assign eligible patients into AT and non-AT groups. The microsimulation analysis was implemented with 50 samplings. Abbreviations: AT = athletic trainer; APAC = The Oregon All Payer All Claims data

We used zip codes to link patients’ medical claims with the enrollment boundaries of Oregon public high schools. To address the data limitation of uncertainty when linking patients to schools that had no ATs (the “non-AT group”) or to schools that had ATs (the “AT group”), we used a microsimulation analysis to include all patients aged 14–18 with AT-relevant claims in the 2011–2014 APAC dataset. Figure 1 illustrates the conceptual model of our microsimulation analysis. We used the geographic correspondence engine known as MABLE/Geocorr 14 (n.d.) to generate the proportion of each zip code’s 2010 census population attributable to the school districts within each zip code. We then calculated the proportion of the zip code’s population within the enrollment boundaries of public high schools that had AT services, ranging from 0 to 1. This was used as an estimate of the probability of a patient in that zip code being assigned to high schools that had AT services. In other words, each patient would be linked by his/her zip code with a probability of being in the AT group, i.e. the probability of receiving AT services.

Because schools could change AT services and patients could move to different zips during the study period, for each patient we summed the payer payments and utilizations across months with the same AT probability, which then entered the microsimulation analysis as a trial. A pseudo-random number ranging from 0 to 1 would be drawn for each trial, and would be compared with the probability of different paths in the decision tree, with higher probability paths being more likely to be selected. In our model (Fig. 1), if the pseudo-random number drawn for a trial fell in the range of being in the AT group, then the payer payments and utilizations would be summed into the AT group; otherwise, they would be summed into the non-AT group.

We used TreeAge Pro 2018 (TreeAge Software, Williamstown, MA) to implement the microsimulation analysis for patients with Medicaid, patients with commercial insurance, and all patients. To address the data limitation of parameters of uncertainty and to stabilize our results, we implemented the microsimulation analysis with multiple samplings for our model. Because results from any single sample of microsimulation may not be stable, we implemented the analysis with 50 samplings, and each sample contained the same number of trials as the original sample. To take into account that schools could change their AT-services and patients could move to different zips during the study period, some patients would be linked with different AT probabilities, resulting in a slightly more trials than patient numbers. In our study, there were 67,889 trials per sample for Medicaid and 91,895 trials per sample for commercial insurance. Therefore, by implementing 50 samplings, we ran a total of approximately 3.4 million and 4.6 million trials for Medicaid and commercial insurance, respectively, leading to stable results and narrow confidence interval. We then used Stata (Release 14. College Station, TX: StataCorp; 2015) to analyze payments and visits and test the difference between non-AT group and AT group based on bootstrap of 50 replications clustered by samples (StataCorp n.d.). Bootstrap is a non-parametric methodology that makes no assumption on normality, can be used for any data distribution, and is a particularly useful alternative to analyze skewed data when other nonparametric rank test may fail (Desgagné et al. 1998). We also described characteristics including age, gender, race, ethnicity, and rurality of zip codes based on the Rural-Urban Commuting Area (RUCA) criteria (University of Washington n.d.). All monetary values were adjusted to 2014 dollars by Consumer Price Index. This study was approved by the Oregon State University Institutional Review Board.

## Results

Our analysis included 64,115 patients with Medicaid and 84,968 patients with commercial insurance who were 14–18 years old and had AT-relevant medical claims from 2011 to 2014. We identified around 320,000 and more than 550,000 visits for Medicaid and commercial insurance, respectively, of which less than 2% were inpatient stays. For patients with Medicaid, about 14% of the visits were emergency department visits, while only about 4% of commercially insured patients’ visits were emergency department visits. As shown in Table 1, the not-AT group and AT group rarely differ in demographic characteristics. For both groups, there were higher percentage of White than black or other race, and the majority were not Hispanic. However, a very high percentage of race and ethnicity were unknown, as many payers did not collect this information. The majority of our samples were living in urban areas for both not-AT group and AT group, and the percentage of urban residence was slightly higher for AT group than not-AT group.

**Table 1:** Descriptive characteristics of microsimulation trials by groups and payer types

	Medicaid		Commercial		Both payer types	
	Non-AT group, *n* = 1,061,823	AT group, *n* = 2,332,627	Non-AT group, *n* = 1,135,575	AT group, *n* = 3,459,175	Non-AT group, *n* = 2,151,531	AT group, *n* = 5,520,007
Age in baseline year 2011, mean (standard error) ^a^	14.5 (0.002)	14.3 (0.001)	14.7 (0.002)	14.4 (0.001)	14.6 (0.001)	14.4 (0.001)
Gender, %						
Female	46.0%	46.3%	46.9%	47.5%	46.5%	47.1%
Male	54.0%	53.7%	53.1%	52.5%	53.5%	52.9%
Race, %						
White	64.4%	59.3%	16.5%	11.8%	38.9%	30.3%
Black	3.8%	3.9%	0.8%	0.9%	2.23%	2.1%
Other	6.4%	6.8%	1.3%	1.0%	3.7%	3.3%
Unknown	25.4%	30.0%	81.5%	86.3%	55.1%	64.4%
Ethnicity, %						
Hispanic	9.1%	12.5%	1.3%	1.2%	5.1%	5.7%
Not Hispanic	56.2%	52.0%	7.9%	6.3%	30.6%	24.1%
Unknown	34.7%	35.5%	90.8%	92.5%	64.3%	70.2%
Residence areas defined by RUCA codes ^b^, %						
Urban area	86.3%	96.3%	89.4%	95.6%	88.0%	99.1%
Rural area	13.4%	0.9%	10.2%	0.9%	11.7%	0.9%
Not defined	0.3%	2.8%	0.4%	3.4%	0.3%	0.0%

^a^Standard errors were estimated based on bootstrap of 50 replications clustered by sample. ^b^Residence areas were defined by the Rural-Urban Commuting Area (RUCA) criteria (University of Washington n.d.)

Abbreviation: *AT* Athletic trainer

With not-AT group and AT group combined, we compared payments and visits between payers, results shown in Table 2. Not surprisingly, commercial insurance had higher payments ($375, *p* < .001), had more overall visits (1.33, *p* < .001), but had less emergency visits (− 0.4, *p* < .001) compared with Medicaid.

**Table 2:** Overall difference in payments and visits between commercial insurance and Medicaid

	Patients with Medicaid	Patients with Commercial Insurance	Difference (95%CI) comparing commercial vs Medicaid	*P* value
Payer payment per patient	$470 ($469, $471)	$845 ($843, $846)	$375 ($373, $376)	<.001
Emergency visits per patient	0.67 (0.66, 0.66)	0.26 (0.26, 0.26)	−0.40 (− 0.40, − 0.40)	<.001
Total visits per patient	4.67 (4.67, 4.68)	6.00 (6.00, 6.01)	1.33 (1.32, 1.34)	<.001

Notes: Bootstrap 95% confidence intervals based on 50 replications clustered by sample are reported in parenthesis. Monetary values were adjusted to 2014 dollars using Consumer Price Index of all items

*Abbreviation*: *AT* Athletic trainer

Table 3 presents the effect of AT services on payments and visits by payer types. Medicaid payments were $450 per patient in the AT group and $514 per patient in the non-AT group. The mean payer payments was significantly higher among Medicaid patients in the non-AT group than in the AT group by $64 (95% CI: $62, $67; *p* < .001). For commercial insurance, our analysis found that the mean payments for the AT groups ($845) was slightly higher than the non-AT group ($842), and the difference was insignificant ($3, *p* = 0.109). In total, the mean payment for all patients with Medicaid and commercial insurance rarely changed when AT services were available (−$1.47, 95% CI: $0.24, −$3.17; *p* = 0.09), because there were 33% more patients with commercial insurance (84,968) than with Medicaid (64,115).

**Table 3:** Effect of AT services on payments and visits by payers types

	Patients with Medicaid	Patients with Commercial Insurance	All patients
Payer payment per patient			
Non-AT group	$514 ($513, $516)	$842 ($839, $846)	$700 ($699, $702)
AT group	$450 ($449, $451)	$845 ($843, $847)	$699 ($698, $700)
Non-AT group vs. AT group	$64 ($62, $67)	-$3 (−$6.83, $0.68)	1.47 (−$0.24, $3.17)
*p* value	<.001	0.109	0.09
Emergency visits per patient			
Non-AT group	0.71 (0.70, 0.71)	0.32 (0.32, 0.32)	0.52 (0.52, 0.52)
AT group	0.65 (0.64, 0.65)	0.25 (0.24, 0.25)	0.41 (0.41, 0.41)
Non-AT group vs. AT group	0.06 (0.06, 0.06)	0.08 (0.07, 0.08)	0.11 (0.10, 0.11)
*p* value	<.001	<.001	<.001
Total visits per patient			
Non-AT group	4.93 (4.92, 4.94)	5.80 (5.79, 5.82)	5.50 (5.49, 5.50)
AT group	4.56 (4.55, 4.56)	6.07 (6.06, 6.08)	5.55 (5.55, 5.56)
Non-AT group vs. AT group	0.37 (0.36, 0.39)	−0.27 (−0.29, −0.25)	−0.06 (− 0.07, − 0.05)
*p* value	<.001	<.001	<.001

Note: Bootstrap 95% confidence intervals based on 50 replications clustered by sample are reported in parenthesis. Monetary values were adjusted to 2014 dollars using Consumer Price Index of all items

*Abbreviation*: *AT* Athletic trainer

Our results suggest that AT services were associated with significantly less emergency visits among both Medicaid and commercially insured patients. Medicaid patients in the AT group had an average of 0.65 emergency visits, significantly less than an average of 0.71 emergency visits among those in the non-AT group (*p* < .001). Patients with commercial insurance in the AT-group also had significantly less emergency visits on average than those in the non-AT group (0.25 vs 0.32, *p* < .001).

However, the results on total visits were different by payer types. As presented in Table 3, Medicaid patients who were in the AT-group had significantly fewer visits on average than those in the non-AT group (4.56 vs 4.93, *p* < .001), or an 8% reduction. By contrast, for the commercially insured patients, AT services were associated with significantly more visits per patient (6.07 vs 5.80, *p* < .001), an increase of 5%.

## Discussion

By analyzing the Oregon APAC data with our microsimulation model, we found significant impacts of high school AT services on medical payments and utilizations. However, the impacts varied by payer types. We propose these differential impacts are mainly driven by the pattern of healthcare utilizations among children with Medicaid and commercial insurance, and by the practice and reimbursement model for high school AT services.

Literature suggests there is a high rate of emergency care utilizations among Medicaid patients (Cunningham 2006; Kim et al. 2017; Tang et al. 2010; Zuckerman and Shen 2004). Children with Medicaid also tend to use emergency department as their major source of care for sports-related injuries. A recent study on concussion visits found that children with Medicaid (37.1%) were far more likely to seek care in the emergency departments than those with private insurance (6.5%) (Arbogast et al. 2016). Another study also suggested that children represented more than 60% of the Medicaid insured patients who used the emergency departments for traumatic brain injury (Selassie et al. 2004). This greater reliance on emergency care among pediatric patients with Medicaid is supported by our findings that children with Medicaid had more than twice the average emergency visits than children with commercial insurance on AT-relevant claims.

ATs in high schools are well-positioned to reduce the utilization of emergency care for athletic injuries through appropriate initial management and early intervention (Fletcher et al. 2014). Given that pediatric emergency visits are more costly for lower acuity cases (Montalbano et al. 2016), AT services in high schools may lead to a significant savings for Medicaid by limiting unnecessary emergency visits. Moreover, it is well documented that children with Medicaid usually experience limited and delayed access to orthopedic care when compared to privately insured children (Kocher et al. 2004; Pierce et al. 2012; Skaggs et al. 2006). Access to AT services allows for early evaluation and timely management of conditions. These early interventions by ATs may aid in resolution of the injury prior to the patient reaching the billable system, which will also contribute to overall costs containment.

Similar to Medicaid insured patients, commercially insured children with access to AT services also exhibited less emergency department visits per patient compared to those without AT services. However, unlike those with Medicaid, our study found a significant increase in overall visits for commercially insured children with AT services. Therefore, despite evidence suggesting that ATs are effectively reducing the number of referrals for costly emergency department visits, children with commercial insurance still exhibit a greater number of total visits compared to commercially insured children without AT services. To explain this phenomenon, the current practice and reimbursement model for AT services may play a key role. High schools in Oregon mainly utilize an outreach model to provide AT services. Under this model, ATs are typically employed and paid by hospitals, physician groups, or physical therapy clinics. These organizations then contract with schools to provide AT services at a reduced fee, and may seek to recoup their cost through increased patient referrals as AT services are generally not recognized for reimbursement purposes by insurance (Kaminski 2017). Therefore, rather than perform services themselves, ATs may be encouraged to refer patients to their hospitals and clinics so that these services can be reimbursed by insurance. Moreover, ATs may be more likely to refer children with commercial insurance than children with Medicaid for a variety of reasons, including higher reimbursement rates, fewer limitations on the types of diagnoses and services eligible for reimbursement, and better compliance with referral due to greater access to care (Pierce et al. 2012; Skaggs et al. 2006). Although it might be expected that the significant increase in overall visits for commercially insured children with AT services would result in greater total costs per patient, the payer payments rarely changed, likely due to the beneficial effects of AT services in reducing expensive emergency department visits.

Our study provides novel evidence for the differential impacts of AT services on children under the current reimbursement model. To inform public policy decisions, we advocate for reforming this model to encourage ATs to work to their highest ability to improve children’s wellbeing while containing avoidable medical payments and utilizations. As our study showed that AT services could save public insurance payments, the legislators and government should consider allocating funds for high schools to directly employ ATs. By replacing the current “clinic outreach model” by a new “school employment model”, we expect that ATs’ incentives to increase referrals will be minimized, and therefore the reduction in medical costs and utilizations will also be realized among children with commercial insurance. Savings of both public and private healthcare dollars will further spur public-private cooperation in financing the school-employment model of AT services (Hambleton et al. 2012). In fact, researchers across the nation have urged all stakeholders to advocate for each state’s funds to support employing ATs in high schools (Hambleton et al. 2012; Mazerolle et al. 2015). We echo this nationwide advocacy by providing evidence from our innovative study.

### Limitation

We recognize several limitations of our study. First, because medical claims data did not contain children’s school attendance information, we were unable to directly link children to specific high schools. The medical claims data also did not contain information that allowed us to limit the claims analyzed to those resulting from high school sport participation only. As a result, it is likely there were a number of claims included in the AT group for which an AT was not actually available to provide service for the injury. While we implemented microsimulation model as an innovative solution to these data limitations, future cohort studies with direct linkage between students and schools can provide stronger evidence. Second, as medical claims did not collect information regarding income, education, and employment, the current data were insufficient for precisely controlling for patients’ socioeconomic status. While we implemented analyses separately for Medicaid and commercial insurance aiming to partially control for patients’ socioeconomic status by their insurance status, this may still limit the strength of our conclusion on AT impacts. Finally, our current retrospective study could not investigate the causal effect of AT services.

## Conclusion

We implemented an innovative microsimulation study on medical claims to examine AT impacts on medical care across years. AT services in high schools could potentially reduce medical payments and utilizations, although the impacts were differential by insurance types under the current reimbursement model. We therefore advocate for public-private cooperation in supporting high schools to directly employ ATs.

## Abbreviations

APAC: All Payer All Claims dataset
AT: Athletic trainer
CI: Confidence interval
CPT: Current Procedural Terminology
ICD9: International Classification of Diseases, 9th edition
